# A new formulation of graphene oxide/fluconazole compound as a promising agent against *Candida albicans*

**DOI:** 10.1007/s40204-019-0109-6

**Published:** 2019-03-11

**Authors:** Sabrieh Asadi Shahi, Shahla Roudbar Mohammadi, Maryam Roudbary, Hamid Delavari

**Affiliations:** 1 Department of Medical Mycology, Faculty of Medical Sciences, Tarbiat Modares University, Tehran, Iran; 2 Department of Medical Mycology and Parasitology, School of Medicine, Iran University of Medical Sciences, Tehran, Iran; 3 Department of Materials Engineering, Tarbiat Modares University, Tehran, Iran

**Keywords:** Antifungal agent, *Candida albicans*, Graphene oxide, Fluconazole

## Abstract

*Candida albicans (C. albicans)* belongs to the opportunistic fungal pathogens, which cause a wide spectrum of infections in immune-compromised patients. Graphene oxide (GO), a biocompatibility agent, has been reported to exhibit effective antimicrobial activity. In the present study, a graphene oxide/fluconazole (GO/Flu) compound was synthesized and characterized using Fourier transform infrared spectroscopy (FTIR) and Raman spectroscopy. The antifungal activity of GO/Flu was examined against fluconazole-resistant *C. albicans* (ATCC 10231) compared to GO and Flu using the broth microdilution method, according to CLSI protocol. DNA fragmentation was assessed through the antifungal mechanism of GO/Flu. The release of Fluin PBS medium was measured. Moreover, the cytotoxicity effect of GO/Flu on SW480 cell line was evaluated. Indeed, adhesion ability of *C. albicans*-treated GO/Flu against SW480 cell line was assessed. The minimum inhibitory concentration (MIC) of GO, Flu, and GO/Flu was determined at 800 µg/mL, 16 µg/mL, and 400–9 µg/mL, respectively. Notably, GO/Flu exhibited an intense antifungal activity compared to GO and Flu. In addition, GO/Flu showed much less cell toxicity against SW480 cell line than GO and Flu (*P* < 0.05). The release determination of Flu in PBS (pH 7.4) medium was 72.42%. GO/Flu reduced the adhesion ability of *C. albicans* to SW480 cell line significantly. DNA fragmentation assay indicated that GO/Flu potentially degraded the DNA of *C. albicans* and caused a fungicidal influence. According to the findings, GO/Flu could enhance the antifungal activity against *C.albicans* through DNA fragmentation with low cytotoxicity effect.

## Introduction

*Candida albicans* (*C. albicans*) is one of the most important opportunistic fungi causing a broad range of diseases from superficial to systemic infections in immunity-compromised patients (Weber et al. 2008; Khan et al. 2003). Among azole antifungal agents, fluconazole (Flu) is an effective and the most common azole for the treatment of candidiasis. Hence, developing drug-resistant *Candida* species can lead to serious therapeutic compliance (Charlier et al. 2006). Recent studies reported the intermediate to high incidence of *Candida* spp. resistant to fluconazole (Casalinuovo et al. 2004). It is well known that various molecular mechanisms are responsible for the development of fluconazole-resistant *C. albicans* (Claudia et al. 2010; Kanafani and Perfect 2008; Alizadeh et al. 2017).

Graphene is characterized as carbon atoms closely packed into honeycomb two-dimensional (2D) lattice possessing unique thermal, mechanical, and electrical properties (Allen et al. 2010). Graphene has a specific high surface area and has a great deal of oxygen bonds in its edges and defective sites such as hydroxyl (C–OH), carboxylic (COOH), carbonyl (C–O), and epoxide groups (C–O–C) accessible on both sides (Stankovich et al. 2007; Haubner et al. 2010). Therefore, graphene due to its potential applications has been remarkably used to construct new composites, particularly nanocomposites such as nanoelectronics, conductive thin films, supercapacitors, biosensors, and nanomedicine approaches (Yang et al. 2012; Shen et al. 2012). The number and range of antifungal drugs are limited and the adverse side effects are still a major therapeutic challenge. Therefore, in the last 5 years, the therapeutic application of graphene oxide (GO) due to its drug delivery characteristics has improved (Sawangphruk et al. 2012). Designing drug delivery systems based on nanocompounds is used to overcome the deficits and disadvantages of conventional pharmaceutical formulations, which is done by reducing the frequency and the amount of drug use which increases the drug’s effect through focusing on the target site (Chaudhary 2013; Alizadeh et al. 2017). Previous studies reported that GO can inhibit the growth of bacterial cells (*Escherichia coli*, *Staphylococcus aureus*) and display superior inactivation effects on copper-resistant bacteria through penetration of GO to bacterial cell membrane (Pasricha et al. 2012; Cong et al. 2010; Liu et al. 2011; Akhavan and Ghaderi 2010). However, prior studies had focused on the antibacterial effect of GO and few relative reports are available about its antifungal activity. Sawangphruk et al. reported that GO inhibits the mycelial growth of *Aspergillus nigger*, *Aspergillus oryzae*, and *Fusarium oxysporum* (Sawangphruk et al. 2012).

GO has emerged support layers that aid in stabilizing, helping Flu to achieve better controlled release and improved antifungal activity. Therefore, nowadays the design of innovative drug delivery strategies for improving the drug release as a novel approach to combat drug resistance is highly deliberated. In the current study, we synthesized GO/Flu compound and evaluated the effect of biogenic GO/Flu against *C. albicans* using the antifungal susceptibility test. Besides, we investigated the cytotoxicity effect of GO/Flu compound against SW480 cell line and DNA fragmentation assay. Our results show that the prepared GO/Flu with a lower concentration can be used instead of Flu and GO.

## Materials and methods

(3-Chloropropyl)triethoxysilane (CPTES), fluconazole powder (Flu), dimethylformamide (DMF), chloramphenicol, MTT(3-(4,5-dimethylthiazol-2yl)-2,5-diphenyltetrazolium bromide), dimethyl sulfoxide (DMSO), Dulbecco’s modified Eagle Medium (DMEM), dialysis bag (a cutoff of 12,000 Da) were purchased from Sigma-Aldrich. Sabouraud dextrose agar media (SDA) (Merck, Germany). Microtiter plates (tissue culture grade, 96 wells, flat bottom, Corning, USA). A standard strain of *C. albicans* (ATCC10231) and SW480 cell line were purchased from the cell bank of the Pasteur Institute of Iran.

### Preparation of GO

GO was synthesized using the Hummers’ method with a minor modification. An improved Hummers’ method without using NaNO_3_ can produce GO in nearly the same level as that prepared by the conventional Hummers’ method (William et al. 1958).

### Structural and morphological characterizations

The morphological and structural characteristics of GO were determined by Fourier transform infrared spectroscopy (FTIR) (Perkin&Elmer—Frontier, USA). The thin plate of FTIR samples was prepared by mixing GO powder and potassium bromide (KBr) which was then compressed under high pressure. The FTIR spectra were measured in the range of 500–4000 cm^−1^. Raman spectra (Almega Thermo Nicolet Dispersive Raman Spectrometer) were recorded in the range of 1000–1700 cm^−1^ with a laser excitation wavelength of 532 nm.

### Synthesis of GO/Flu

For this purpose, 2.4 mg of GO was dispersed in 24 mL toluene through sonication to achieve a homogeneous GO suspension (final concentration is 0.5 mg/mL). The UV absorption of the supernatant was studied. (3-Chloropropyl)triethoxysilane (CPTES) (Sigma-Aldrich) was added into the reaction and was sonicated for 30 min. The mixture was heat-treated in an oven at 60 °C for 6 h. The product was centrifuged at 1000 rpm for 5 min and washed with methanol twice to remove the impurities. Finally, GO–Cl (the free chlorine remaining in the CPTES structure after binding with GO, which contributes to the binding of Flu) was obtained. For the synthesis of GO/Flu compound, 108 µg of Flu (Sigma-Aldrich) was added to a GO–Cl suspension prepared in the previous step dispersed in 9 mL dimethylformamide (DMF) (Sigma-Aldrich). The reaction mixture was refluxed at 140 °C in an oil bath for 24 h. The solid phase was centrifuged at 1000 rpm for 5 min and washed with DMF. The final product was thoroughly washed with water to reach pH 7.4.

### Confirmation of flu loading on GO–Cl

The amount of loaded Flu was determined using the standard UV absorption curve in different concentrations of Flu (1.05–108 µg/mL) at 260 nm. Besides, we investigated the absorption of GO–Cl–Flu supernatant against GO–Cl supernatant.

### In vitro Flu release

The release rate of Flu from GO/Flu was compared to pure commercial Flu. Separately, for this purpose, 500 μL of GO/Flu (400/9 µg/mL) and 500 µL of Flu (9 µg/mL) (the initial concentration of Flu in both samples, i.e., pure Flu and GO/Flu are same as 9 µg/mL) were released using a dialysis bag (cutoff 12,000 Da, Sigma) in 15 mL PBS buffer (pH 7.4). At 45 min interval for every 2 min, 1.5 mL of buffer was removed and its concentration was measured using a spectrophotometer at 260 nm wavelength. This amount was then re-entered into the original buffer. The release of Flu in PBS (pH 7.4) medium was determined at 72.42%. The experiment was performed at least three times.

### Antifungal study

The minimum inhibitory concentration (MIC) of GO, Flu, and GO/Flu compound was examined using the microdilution broth according to the CLSI method (2002). A standard strain of *C.albicans* (ATCC10231) was cultured on Sabouraud dextrose agar (SDA, Merck, Germany) containing chloramphenicol (sigma) and was incubated at 35 °C for 24 h. After that, 1 × 10^3^ CFU/mL *Candida* suspension cell was prepared. Drug susceptibility test was performed in sterile U-bottomed 96-well microtiter plates. For this, 100 Âµl Flu (1–128 μg/mL), GO (12.5–1600 μg/mL), 50 μL of Flu, and 50 μL of GO were added into eight columns and eight rows of microtiter plate at series of concentrations. Also, 100 μL of Flu and GO were added separately to each well containing RPMI medium at series of concentrations. Finally, 100 μL of yeast suspension was added to each well and were placed on shaker for 3–5 min and were incubated at 37 °C for 24 h. Each experiment was repeated three times in an independent manner. Moreover, proper positive and negative control was used. The minimum concentration of drug that inhibited the *Candida* growth was described as MIC.

The minimum fungicidal concentration (MFC) was determined as the minimum concentration of drug that led to death of fungi and followed by culturing fungi on SDA.

FIC < 5; synergistic effect

0/5 < FIC < 1 relative synergistic effect

FIC = 1 additive effect

1 < FIC < 4 indifferent

FIC > 4 antagonistic effect

FIC: fractional inhibitory concentration

$${\text{FIC}}\, = \,\left( {{\text{MIC combination A}}/{\text{MIC Alone A}}} \right)\, + \,\left( {{\text{MIC combination B}}/{\text{MIC Alone B}}} \right)$$

### Cytotoxicity of GO, Flu and GO/Flu against SW480 cell line

Cytotoxicity of GO, Flu, and GO/Flu compounds were evaluated through MTT assay against SW480 cell line (Roudbary et al. 2015). Briefly, 100 µL of RPMI containing 1 × 10^3^ cell suspension was seeded in each well of microtiter plates (tissue culture grade, 96 wells, flat bottom, Corning, USA). After 24 h, 100 µL of Flu (16 µg/mL), GO (800 µg/mL), and GO/Flu (400/9 µg/mL) (GO at 400 µg/mL concentration and Flu at 9 µg/mL concentration) were added and incubated in 5.0% CO_2_ incubator in 37 °C for 24 h. The cell suspension without any treatment was considered as the control group. The array was performed triplicate in each experiment. After the incubation time, 20 µL of MTT (3-(4,5-dimethylthiazol-2yl)-2,5-diphenyltetrazolium bromide) (Sigma, 5 mg/mL in PBS) was added in each well. After 4 h incubation under the same condition, the supernatant was removed and 30 µL dimethyl sulfoxide (DMSO) was added to each well. When the purple formazan crystals were dissolved completely after 10 min of mild shaking, the spectrophotometrical absorbance was measured in microtiter plate (state fax 2100 microplate reader) reader at 590 nm wavelength. The cell viability was calculated using the following formula:

Cell viability = OD test/OD control (absorption of positive control (SW480 cells and medium) × 100

### Adhesion assays

To evaluate the effect of GO/Flu compound on *Candida* ability to adherence to the SW480 cells, fungal cells were treated with different concentrations of Flu (16 µg/mL), GO (800 µg/mL) and GO/Flu (400/9 µg/mL) and were co-cultured with SW480 cells. For this purpose, 1 × 10^3^ fungal suspension and 100 μL of SW4801 × 10^3^ were prepared in a Dulbecco’s Modified Eagle Medium (DMEM) medium and seeded in microplate wells, and were then incubated at 37 °C for 24 h. The supernatant containing non-adherent *Candida* cells was removed and 10 μL of the suspension containing adherent *Candida* cells was cultured on Sabouraud dextrose agar (SDA, Merck). After 24 h incubation at 35 °C, the colony count was performed (Silva-Dias et al. 2015). Each experiment was performed at least three times.

### DNA fragmentation assay

For determining DNA fragmentation, *C.albicans* was treated with MIC concentration of GO/Flu compound and then the genomic DNA of treated and non-treated *C.albicans* was extracted using phenol/chloroform reagent, glass bead, and lysis buffer, as described previously (Roudbary et al. 2012).

The digested fragments were electrophoresed through 1.8% agarose gel and then were visualized using ethidium bromides staining.

### Statistical analysis

MTT tests were analyzed using one-way analysis of variance (ANOVA) and Tukey tests using SPSS software, version 20 (SPSS, Chicago, IL, USA). Each experiment was performed at least three times. Variations in the colony count among treatment groups in adhesion assays were assessed using the Pearson’s Chi square or Fisher’s exact test. Variations in the colony count between the studied groups were assessed using the Mann–Whitney *U* test. A *p* value of ≤ 0.05 was considered to be statistically significant.

## Results and discussion

### Characterization of GO and GO/Flu compound

**Fig. 1:**
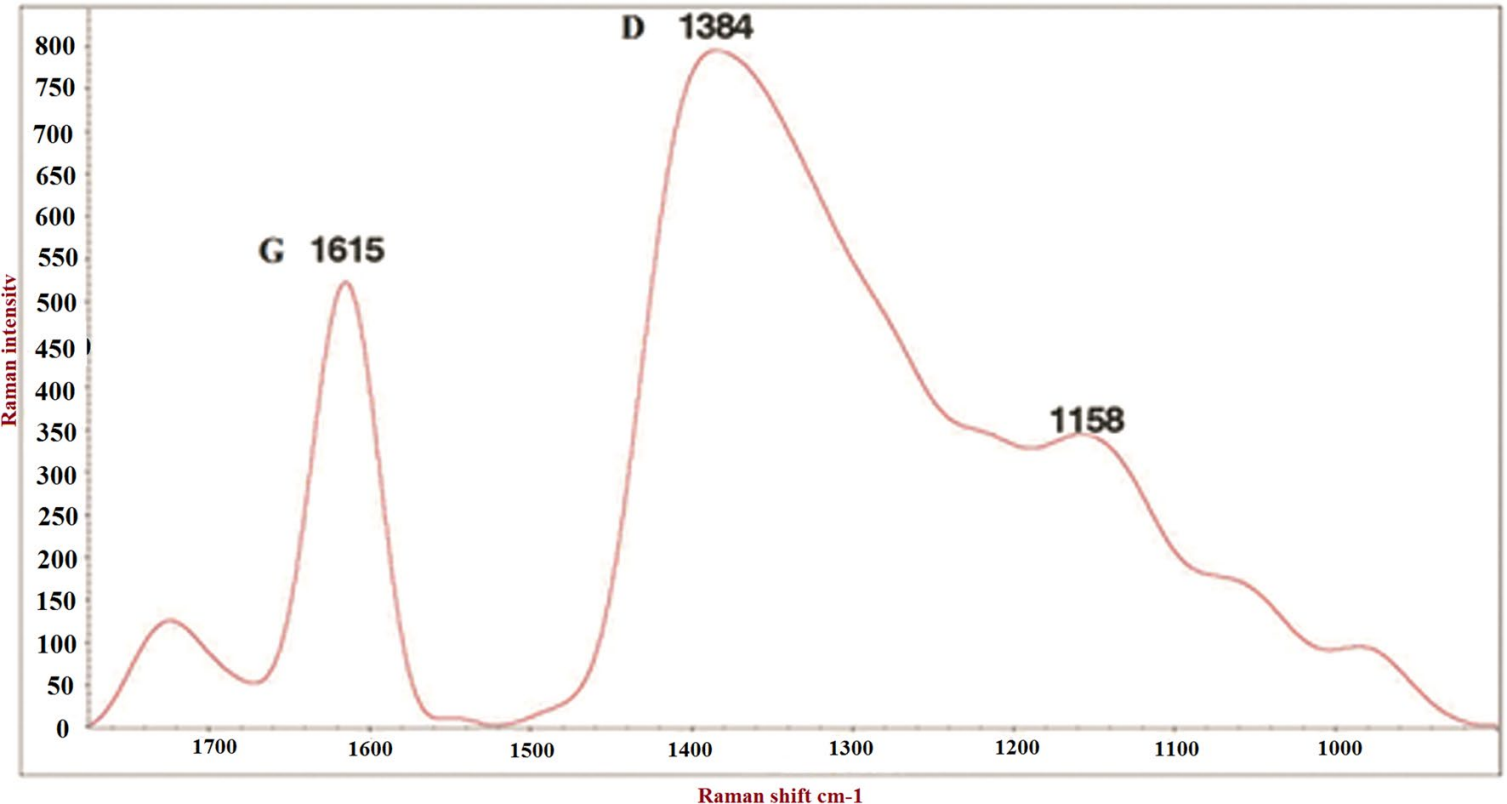
Raman spectrum of GO in the range of 1000–1700 cm^−1^

The GO Raman spectrum confirmed G and D bands at 1615 and 1384 cm^−1^, respectively (Fig. 1). In a Raman spectrum for carbon materials, the G band is a characteristic feature of the graphitic layers and corresponds to the tangential vibration of the carbon atoms, while the D band corresponds to the disordered carbon or defective graphitic structures. The integral intensity ratio of these two peaks scales with the degree of graphitic ordering of the carbons. According to the reference peak, D and G bands indicated the GO structure was regular and consistent.

**Fig. 2:**
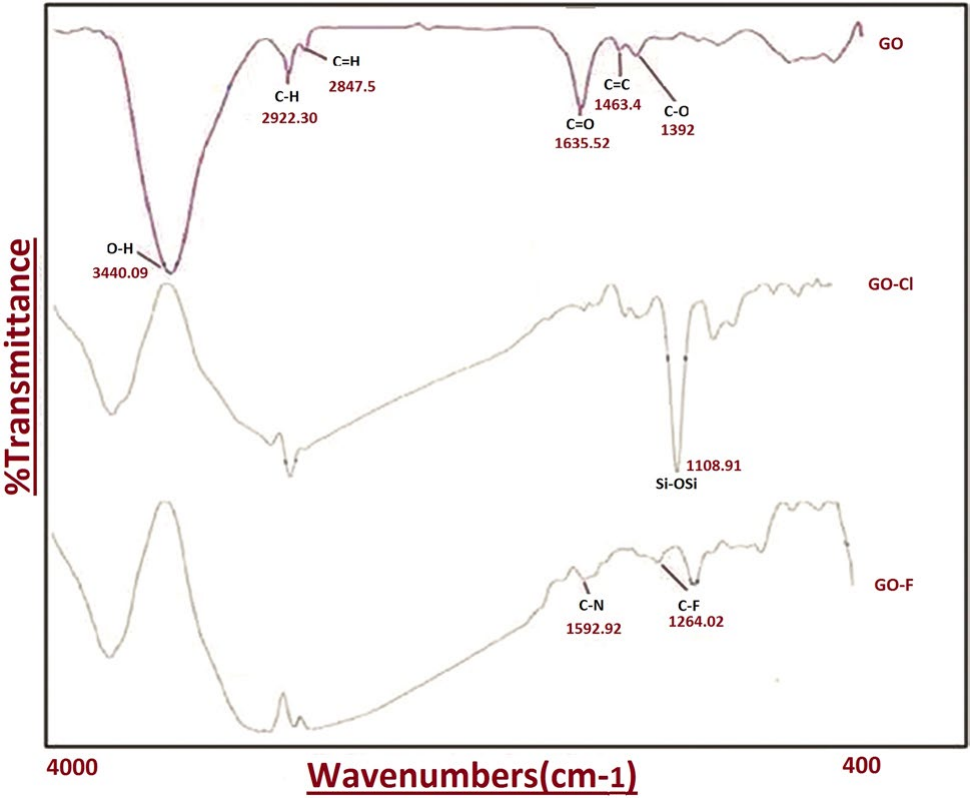
FTIR spectra of GO, GO/Flu and GO/Flu compound

To confirm the structure of GO, GO/Flu, and Flu loading GO with CPTES, FTIR assay was carried out. In the functional GO, the groups are OH at 3500 cm^−1^, C–O at 1084 cm^−1^, C=O at 1726 cm^−1^, and C=C at 1627 cm^−1^. Moreover, GO–Cl indicated that SiOCH_3_ group of CPTES and OH group of GO were combined successfully and the methyl group was deleted. In addition, O of GO with Si of the linker produced SiOSi (the peak is represented at 1108 cm^−1^). The GO/Flu combination showed 1592 and 1264 peaks that were made of C–N in GO and C–F of Flu groups, respectively. The FTIR spectra of GO, GO–Cl, and GO/Flu compound are shown in Fig. 2. The results showed that GO with a suitable plate structure have hydrophilic, hydroxyl, and acidic (C=C, C=O, C–O, O–H) groups. Therefore, this substrate was approved as a proper carrier with antifungal activity.

### Confirmation of Flu loading on GO–Cl

**Fig. 3:**
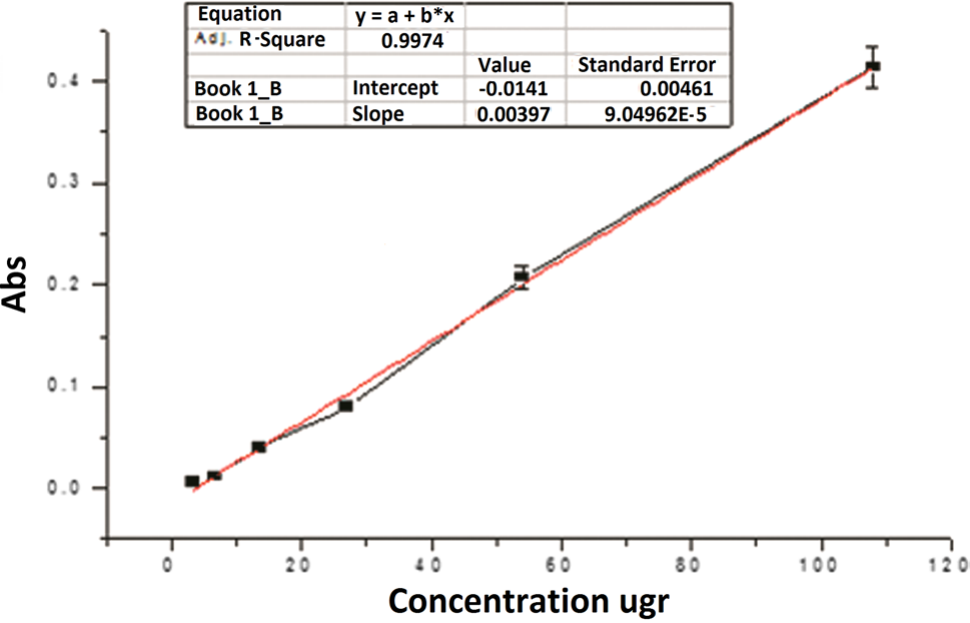
Flu calibration in DMF with pH 7.4

The total amount of Flu that loaded on GO was determined to be 53.42% by the calibration curve of Flu. The amount of Flu loaded on GO was approximately half of the total Flu used in the combination (42.93 μg/mL) (Fig. 3).

### Antifungal activity of GO, Flu and GO/Flu

MICs of GO and Flu against the *C.albicans* were 800 µg/mL and16 µg/mL, respectively. The MIC of GO/Flu compound was 400/9 µg/mL, while GO at 400 µg/mL concentration and Flu at 9 µg/mL concentration showed a high synergistic effect (FIC = 1). Based on the MIC findings, the antifungal properties of GO were remarkable. It could be postulated that interaction of hydrophilic feature GO and hydrophobic *Candida* cell wall has increased. The MIC of GO/Flu compound indicated that its antifungal activity is less than the commercial Flu. This decrease at MIC concentration may be related to the increased release of Flu in the medium. The antifungal properties of GO may be related to some probable mechanisms. First, the sharp edges of the plates affect the cell membrane and lead to the lysis of the cell. Second, through chemical oxidation, the membrane pump disrupts the cell and eventually leads to fungal cell splitting as well as reactive oxygen species (ROS) production causing cell death (Buzea et al. 2007). However, the main mechanism of nanomaterials exploitation is not well known yet, while various in in vivo and in vitro studies suggest that they are able to produce ROS and thus contribute to the accumulation of intracellular calcium, activation of transcription factors, and changes in cytokines (Uusitalo and Hempel 2012). Previous studies are in agreement with our findings that directed the effect of GO on fungi and bacteria with possible mechanisms of action. Chao Li et al. (2013) explored the antifungal activity (AgNPs–CNSS) against *C.albicans* and *C. tropicalis* where MIC GO containing nanosilver particles was significantly less than silver (Li et al. 2013).

Johnny Chen et al. (2013) examined the mechanism of interaction of GO with *F. graminearum* and *F. oxysporum* cell wall which showed that GO disrupted the cell membrane and resulted in electrolyte leakage (Chen et al. 2014). Azimi et al. evaluated the antimicrobial activity of nanoparticles of GO functionalized with hydrophilic chlorophyllin extracted from spinach leaves against *Escherichia coli* (Azimi et al. 2014). Savganfrak et al. (2012) investigated the fungicidal properties of revived rGO, GO on *A. nigger, A. oryzae, F. oxysporum* and reported the IC50 of about 100 μg/mL (Sawangphruk et al. 2012).

### In vitro release of Flu

**Fig. 4:**
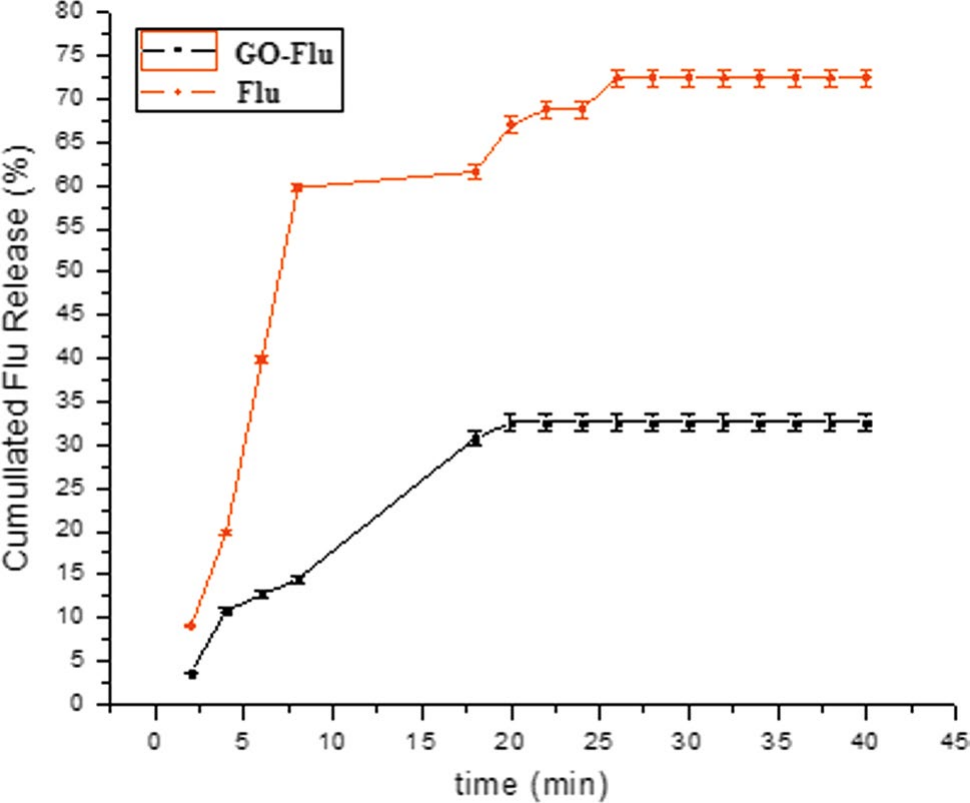
The rate of Flu release from GO/Flu compared to commercial Flu in PBS buffer at pH = 7.4

The release of Flu and Flu from GO/Flu compound in a PBS medium (pH 7.4) was evaluated. Flu calibration chart was prepared in PBS. Then, the concentrations were evaluated according to the calibration chart. The results displayed that the amount of Flu alone released after 6 min was 39.82% and after 45 min was 72.42%, while the release of Flu from GO/Flu was 12.65% after 6 min and 32.58% after 45 min (Fig. 4). Flu was released from GO/Flu to provide the minimum amount needed for the effectiveness of synergism on *Candida albicans*.

### MTT-based cytotoxicity

**Fig. 5:**
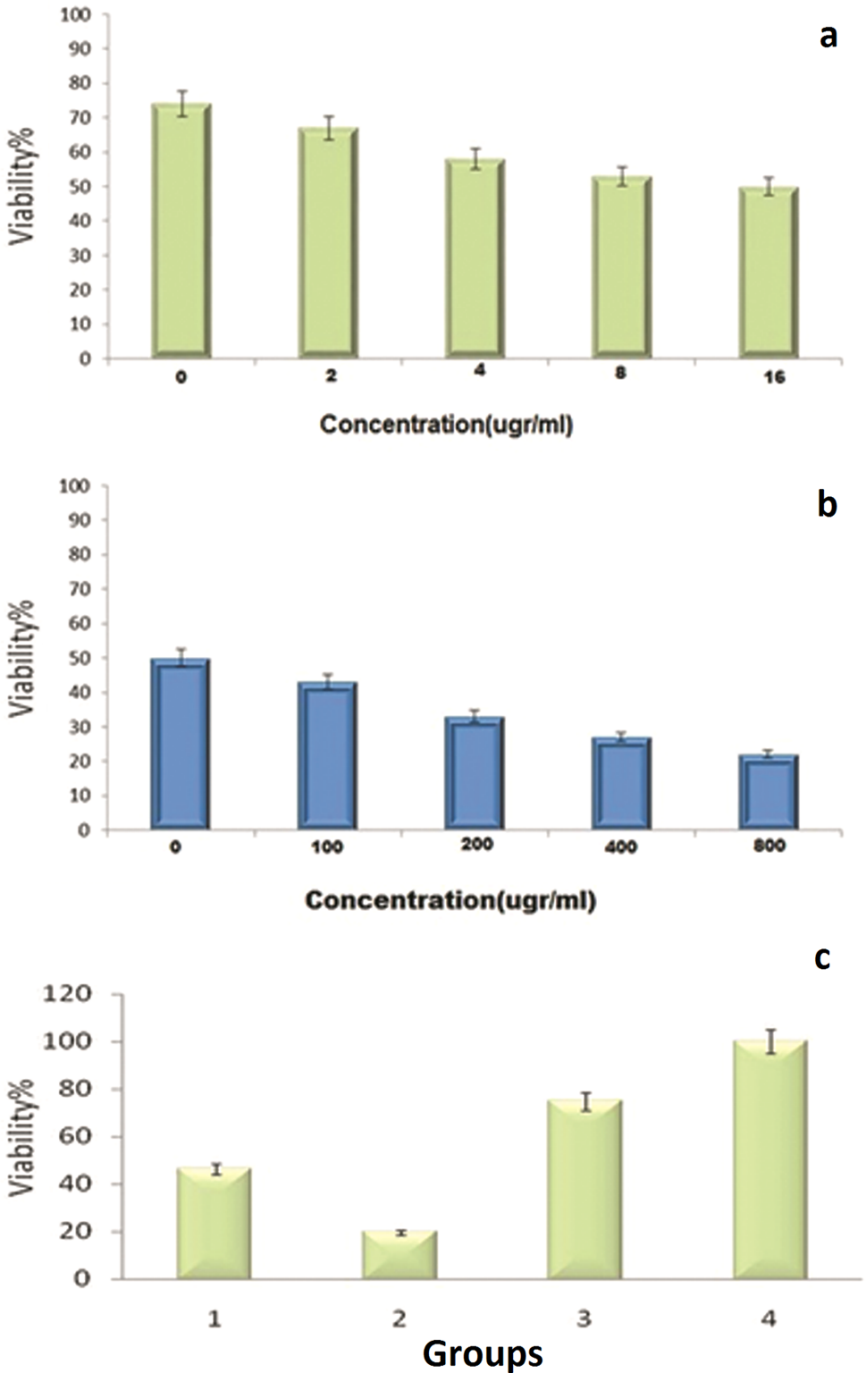
**a** The viability of SW480 cells treated with commercial Flu at different concentrations (0, 2, 4, 8 and 16 μg/mL). **b** The viability of SW480 cells treated with different concentrations of GO. **c** The comparison of viability of SW480 cells treated with (1) Flu (concentration 16 μg/mL); (2) GO (800 μg/mL);(3) GO/Flu compound (400–9 μg/mL; (4) and untreated cells (control group)

GO/Flu showed a dose-dependent manner on the cell viability of SW480 cell line and much less toxicity compared to GO and Flu. The cell viability was reduced to 20% for cells treated with GO at 800 µg/mL and to 46% for Flu treatment cells at 16 µg/mL concentration (Fig. 5). The viability of SW480 cells against Flu and GO is shown in Fig. 3a, b, respectively. Our findings showed that the toxic activity of agents was dose-dependent. Notably, at high concentrations of GO and Flu, there was a significant cell death, while at lower concentrations, a minor cytotoxicity was detected; thus, the difference between groups was significant (*P* < 0.05). Interestingly, GO exhibited higher cell toxicity than Flu; nevertheless, the toxicity decreased significantly in GO/Flu-treated cells compared to the other group. In contrast with our results, Yanli Chang et al. (2011) explored the toxicity of GO on A549 cell line and showed that 67% of cells survived at a concentration of 200 μg/mL (Chang et al. 2011). Chao Li (2013) also reported 60% and 20% cytotoxicity of GO–AgNPs and GO, respectively, on GES-1 at a concentration of 100 μg/mL using the MTT method (Li et al. 2013).

### Adhesion assays

**Fig. 6:**
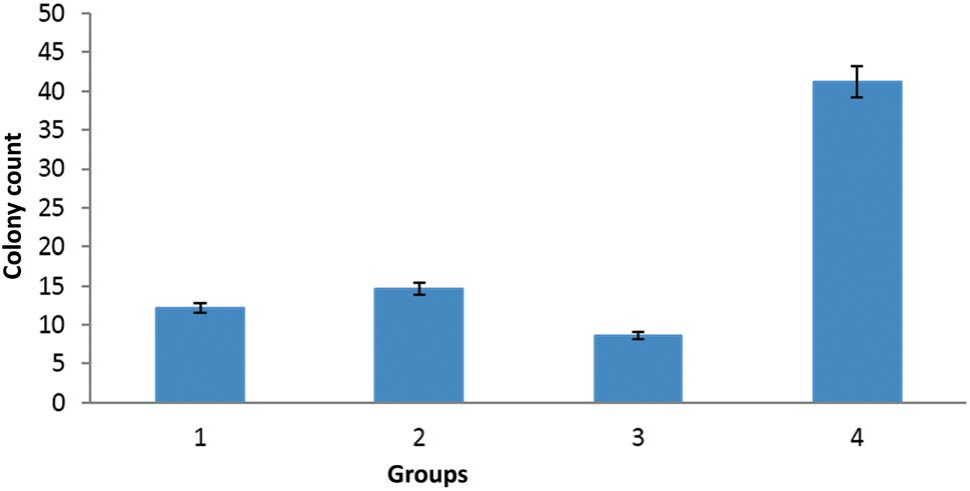
SW480 cells were co-cultured with *Candida* that treated by Flu, GO, GO/Flu previously. The supernatant contains non-adherent *Candida* cells were removed, subsequenty adherent *Candida* cells cultured on SDA medium. 1: Flu (concentration 16 µg/mL); 2: GO (800 µg/mL); 3: GO/Flu (400–9 µg/ml) with GO at 400 µg/ml concentration and Flu at 9 µg/ml concentration); 4: control

As shown in Fig. 6, the lowest number of *candida* colonies was found in the GO/Flu group. Variations in the colony count between the studied groups were assessed using the Mann–Whitney *U* test. Indeed, in the adhesion assays, a significant difference was found between the number of *Candida* colonies in the Flu, GO, and GO/Flu treatment cells and the control group (*P* < 0.05). Remarkably, we found that *Candida* colonies treatment with GO/Flu was lower than that of GO and Flu (*P* < 0.05). It can be concluded that GO/Flu strongly inhibited *Candida* adhesion to SW480 cells. It may be explained that the compound affected the genes responsible for *C. albicans* attachment. It is well established that *Candida* attachment is the first step for inducing the pathogenesis; therefore, GO/Flu inhibits the successful adhesion of *Candida* to cells and can be a proper agent to prevent the *Candida* attachment.

### DNA fragmentation

**Fig. 7:**
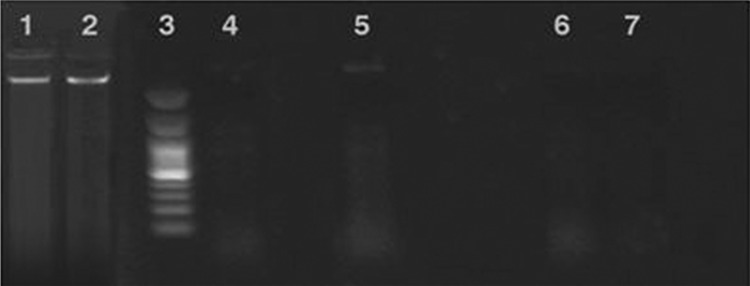
Electrophoresis image from genomic DNA *Candida* in different treatment groups. (1, 2) *Candida* non-treatment, (3) marker 100 bp, (4) GO treatment, (5) Flu treatment, (6) GO/Flu compound treatment, (7) DNA treated with DNase

As shown in Fig. 7, the DNA fragmentation test indicated that GO/Flu notably degraded the *Candida* DNA when compared to Flu, GO, and control groups. The result of DNA fragmentation showed that DNA of *C. albicans* treated with GO/Flu was much more degraded than other treated groups (Kim et al. 2011; Singh et al. 2009; Pisanic et al. 2007). Taken together, the obtained results disclosed the proper anti-*Candida* properties of GO/Flu. Furthermore, this compound has a low cytotoxicity effect on the cell line which ensures its safety regarding human cells.

## Conclusion

The obtained findings suggest that the synthesized GO/Flu compound exhibits appropriate antifungal activity against *C. albicans* and that its capacity had been increased with synergistic effect. As this compound showed no significant toxicity against SW480 cells, therefore it is noticed as a safe agent against human cells. Collectively, this compound could be used as a proper candidate for a therapeutic approach against candidiasis as well; however, a comprehensive in vitro and in vivo study is required in the future.
